# Increased first and second pulse harmonics in Tai Chi Chuan practitioners

**DOI:** 10.1186/s12906-016-1058-4

**Published:** 2016-02-29

**Authors:** Wan-An Lu, Yung-Sheng Chen, Cheng-Deng Kuo

**Affiliations:** Laboratory of Biophysics, Department of Medical Research, Taipei Veterans General Hospital, Taipei, 112 Taiwan; Institute of Cultural Asset and Reinvention, Fo-Guang University, Yilan, 262 Taiwan; Department of Exercise and Health Sciences, University of Taipei, Taipei, 111 Taiwan; Division of Respiratory Care, Department of Chest Medicine, Taipei Veterans General Hospital, Taipei, 112 Taiwan

## Abstract

**Background:**

Tai Chi Chuan (TCC) is known to be a good calisthenics for people. This study examined the relationship between pulse harmonics and autonomic nervous modulation in TCC practitioners.

**Methods:**

Power spectral measures of right pulse wave and heart rate variability (HRV) measures were compared between TCC practitioners and control subjects. Correlation analyses between pulse harmonics and HRV measures were performed using linear regression analysis.

**Results:**

At baseline, the total power of pulse (TPp), powers of all individual pulse harmonics, normalized power of the 1^st^ harmonics (nPh1) of TCC practitioners were greater, while the normalized power of the 4^th^ pulse harmonics (nPh4) of TCC practitioners was smaller, than those of the controls. Similarly, the baseline standard deviation (SD_RR_), coefficient of variation (CV_RR_), and normalized high-frequency power (nHFP) of RR intervals were smaller, while the normalized very low-frequency power (nVLFP) and low-/high- frequency power ratio (LHR) were larger in the TCC practitioners. The TCC age correlated significantly and negatively with nPh1, and nearly significantly and negatively with nPh2 in the TCC practitioners. Thirty min after TCC exercise, the percentage changes in mRRI, SD_RR_, TP, VLFP were decreased, while the percentage changes in HR, ULFP, nLFP, and Ph2 were increased, relative to the controls. Correlation analysis shows that the %Ph2 correlates significantly and negatively with %mRRI and significantly and positively with %HR.

**Conclusion:**

The TCC practitioners had increased baseline total power of pulse and the 1^st^ and 2^nd^ pulse harmonics, and decreased power of the 4^th^ pulse harmonics, along with decreased vagal modulation and increased sympathetic modulation. After TCC exercise, the power of the 2^nd^ harmonics of TCC practitioners was increased which might be related to the increase in HR due to decreased vascular resistance after TCC exercise.

## Background

Arterial pulse is one of the vital signs in clinical medicine, and has since antiquity been used by the physician as one of the arts of medicine [1]. Since the pulse wave travels outwards from the heart to other organs and tissues, the arterial pulse wave can be influenced by not only the condition of the heart, but also the conditions of the nerves, muscles, skin, arterial walls, and blood constituents. Arterial pulse wave can thus reflect the rhythmic contractility of the heart, the tension of the vessel wall, and the smoothness of the blood vessels [2]. Many studies have demonstrated the usefulness of pulse waveform analysis in clinical medicine [3–13]. For instance, the volume and contour of arterial pulse reflect a combination of cardiovascular functions [3]; the radial waveform can describe the natural history of essential hypertension, the difference between hypertension and chronic nephritis, and the effects of arterial degeneration with ageing on the arterial pulse [9, 10]. Recent investigations also demonstrated that the heart rate variability (HRV) is decreased in many kinds of clinical diseases [6–8].

Several pioneer researchers have used Fourier transform in the analysis of femoral artery pressure gradient, arterial input impedance and flow waveforms [4, 11–13]. By using Fourier transform and a small balloon to simulate an organ (group of arterioles) attached to the aortic trunk, it was found that the aorta and the closely attached organ can produce coupled oscillation analogous to a resonance circuit [14–17]. The capacitance or the ability of the attached organs to hold blood was shown to be related to specific Fourier components of the blood pressure pulse via their influence on the blood pressure wave propagation, and thus the blood distribution to the body [14–17]. It has also been shown that the power of harmonics of arterial pulse wave can be described by an exponential decay function with respect to the order of harmonics in both non-pregnant and pregnant women [18]. Therefore, the power spectral analysis of the pulse waveform might be a useful method with which to assess the cardiovascular function of the subject [5].

For the elderly, the low-velocity and low-impact exercises are preferred to reduce their cardiovascular and orthopedic complications [19]. Goble et al. [20] reported that lower-intensity exercise might have similar training effects as higher-intensity exercise for cardiac patients. For patients with acute myocardial infarction referred for cardiac rehabilitation, low- and high-intensity exercise training have been shown to improve the functional capacity and produce relatively similar changes in cardiorespiratory variables during the initial 3 months of exercise training [21]. Tai Chi Chuan (TCC) is a traditional oriental conditioning exercise or calisthenics that can defer the age-related decline in aerobic capacity [22]. TCC training has been shown to be beneficial to the cardiopulmonary function [23, 24], balance [25], and strength [24], and can reduce the tension, anxiety, and mood disturbance [26] of the subjects. Since there are more than one hundred million TCC practitioners in the world [27], it is necessary to understand the physiological effects of TCC on the subjects.

The aim of this study was to examine the effect of TCC on the pulse harmonics of arterial pulse wave and the relation between the pulse harmonics and the autonomic nervous modulation of TCC practitioners.

## Methods

### Study subjects and study design

Both TCC practitioners and sedentary subjects without TCC experience were included in this study. Thirty TCC practitioners (19 male and 11 female) with TCC training age over 1 year and thirty healthy sedentary subjects (14 male and 16 female) were recruited from a TCC training center and community, respectively. The Institute Review Board of the Taipei Veterans General Hospital has approved this study (VGHIRB No. 97-10-25A). The procedure was fully explained to the subject and informed written consent was obtained from the subject before the study. The subject who had major cardiopulmonary disease or was on regular medicine for diabetes mellitus, hypertension, renal or liver disease was excluded from the study.

### Equipment

The subject included in this study was instructed not to take caffeinated or alcoholic beverages for at least 24 h prior to study. Before TCC, the subject was instructed to lie down and take a rest in supine position. After 5 min' rest, the trends of conventional ECG signals and pulse waves signals from the index finger of right hand of the subject were picked up by the PowerLab 16sp (ML795 PowerLab/16sp, ADInstruments, Sydney, Australia) and transferred to a laptop for 10 min. The Quad Bridge Amp and PowerLab/16sp Amplifier (ADInstruments Inc, Australia) were used to amplify the signals, and PowerLab Chart 4.2 software was used to record the pulse signals. The sampling frequency for data acquisition was 400 Hz.

After baseline recording, the TCC practitioners were advised to exercise classical Yang style TCC for 40 min. Each session of Yang's TCC included 10 min' warm-up exercise (lower back and hamstring stretching, gentle calisthenics, and balance training), 20 min' TCC exercise, and 10 min' cool-down exercise. Each set of Yang's TCC included 64 postures [27]. During TCC exercise, the subject kept the same pace in practicing the postures of TCC in sequence by performing those postures according to a pre-recorded tape to ensure that the same pace and sequence of postures were followed by the TCC practitioners. Thirty minutes after the completion of TCC exercise, the second trends of ECG signals and pulse waves were recorded using the same methodology. All procedures were performed in a bright and quiet room with a constant temperature of 24 to 25 °C.

The same procedures were applied to the control subjects except that the practice of TCC was replaced by resting for the same period of time.

### Spectral analysis of plethysmographic waveform

A total of 215 pulse signal data points (amounting to 81.92 s) from the right index finger were used for power spectral analysis using fast Fourier transformation (Mathcad 11, Mathsoft Inc, Cambridge, MA, USA). Direct current component was excluded before power spectral analysis. The major peak located around the heart rate in the spectrum was identified as the fundamental frequency and termed the 1^st^ harmonic. Subsequent serial peaks located at multiples of the fundamental frequency were identified and termed from 2^nd^ to the 8^th^ harmonics. Beyond the 8^th^ harmonic, the spectral peaks were too small to discern. Hence, only 8 harmonics were included in the statistical analysis. The following terms and definitions were used in this study: Fx, frequency of x^th^ harmonic; nPhx, normalized power of the x^th^ harmonic (nPhx = Phx · 100/TP; Phx, power of x^th^ harmonic; TPp, total power of pulse in the power spectrum). Since the frequency of each harmonic is the multiple of the heart rate which is the fundamental frequency [28], only the frequency of the 1^st^ harmonics was compared in this study.

### Spectral heart rate variability (HRV) analysis

The R-wave detecting software was developed to identify the peaks of the R waves in the recorded ECG signals and to measure the consecutive RR intervals. The last 512 stationary RR intervals were used for power spectral analysis of HRV. Both atrial and ventricular ectopic beats were eliminated before time and frequency domains HRV analyses. If the percentage of ectopic beats were greater than 5 %, the subject was excluded from statistical analysis.

The mean RRI (mRRI), heart rate (HR), standard deviation (SD_RR_), coefficient of variation (CV_RR_) of the last 512 stationary RR intervals were calculated using standard formulae for each subject. The power spectra of 512 RR intervals from ECG were obtained by means of fast Fourier transformation (Mathcad 11, Mathsoft Inc., Cambridge, MA, USA). Direct current component was excluded before the calculation of the powers. The area-under-the-curve of the spectral peaks within the range of 0.01–0.4 Hz was defined by total power (TP). The area-under-the-curve of the spectral peaks within the ranges of 0–0.01 Hz, 0.01–0.04 Hz, 0.04–0.15 Hz, 0.15–0.40 Hz were defined as the ultralow-frequency power (ULFP), very low-frequency power (VLFP), low-frequency power (LFP), and high-frequency power (HFP), respectively.

The Task Force of the European Society of Cardiology and the North American Society of Pacing Electrophysiology has suggested that the power within the frequency range of 0.04–0.4 Hz be used for the normalization of LFP and HFP [29]. In this study, the normalized high-frequency power (nHFP = HFP/TP) was used as the index of vagal modulation, the normalized low-frequency power (nLFP = LFP/TP) as the index of sympathetic and vagal modulations, the low-/high-frequency power ratio (LHR = LFP/HFP) as the index of sympathovagal balance [30], and the normalized very low frequency power (nVLFP = VLFP/TP) as the index of renin-angiotensin-aldosterone system and vagal withdrawal [31].

### Statistical analysis

Data are presented as mean ± SD or median and interquartile range (25^th^ to 75^th^ percentiles) values, depending on the normality of the data, which was determined by the Kolmogorov-Smirnov test. The Mann-Whitney rank sum test was employed to compare the baseline characteristics, pulse spectral indices and HRV measures between TCC practitioners and healthy controls. The Wilcoxon signed rank test was performed to compare the pulse spectral indices and HRV measures before TCC or resting and 30 min after TCC or resting. A *p* < 0.05 was considered statistically significant. All statistical analyses were performed using SigmaPlot 13 software (Systat Software Inc. San Jose, CA 95110, USA).

To quantify the effect of TCC on the autonomic nervous modulation, the percentage changes in spectral indices and HRV measures after TCC or resting in each subject were calculated using the following formula:

$$ \%\mathrm{X}=\left[\left({\mathrm{X}}_{30\  \min\ \mathrm{after}\ \mathrm{T}\mathrm{C}\mathrm{C}}-{\mathrm{X}}_{\mathrm{before}\ \mathrm{T}\mathrm{C}\mathrm{C}}\right)/\left({\mathrm{X}}_{\mathrm{before}\ \mathrm{T}\mathrm{C}\mathrm{C}}\right)\right]\times 100, $$

where the “X” stands for the variable to be compared.

## Results

### General characteristics at baseline and 30 min after resting or TCC exercise

The general data of these subjects are shown in Table 1. There were no significant differences in the baseline characteristics between the controls and TCC practitioners. Similarly, there was no significant difference in blood pressures between baseline and 30 min after TCC or resting in the control and TCC groups.

**Table 1:** Basic characteristics of the controls and TCC practitioners

	Control	TCC	P value
	(n = 30)	(n = 30)	
*General Characteristics*			
Gender (M/F)	14/16	19/11	1.000
Age (year)	49.9 ± 9.4	53.6 ± 10.7	0.198
Height (cm)	163.7 ± 9.3	163.7 ± 10.1	0.790
Weight (kg)	63.9 ± 10.8	63.0 ± 12.2	0.813
BMI (kg/m^2^)	23.7 ± 2.6	23.4 ± 3.0	0.460
TCC age (year)	0	7.0 ± 8.2	NA
*Baseline*			
SBP (mmHg)	119.3 ± 14.9	119.0 ± 12.7	0.947
DBP (mmHg)	68.7 ± 10.4	72.8 ± 8.9	0.089
MABP (mmHg)	85.6 ± 10.8	88.2 ± 9.1	0.258
PP (mmHg)	50.5 ± 11.6	46.2 ± 10.5	0.139
*30 min after TCC*			
SBP (mmHg)	119.1 ± 14.4	114.6 ± 12.8	0.363
DBP (mmHg)	72.4 ± 9.9	72.1 ± 7.8	0.802
MABP (mmHg)	88.0 ± 10.7	86.3 ± 8.2	0.657
PP (mmHg)	46.7 ± 9.4	42.5 ± 11.0	0.098

Data presented are Mean ± SD. *SBP* systolic blood pressure; *DBP* diastolic blood pressure; *MABP* mean arterial blood pressure; *PP* pulse pressure; NA not assessed

**Fig. 1:**
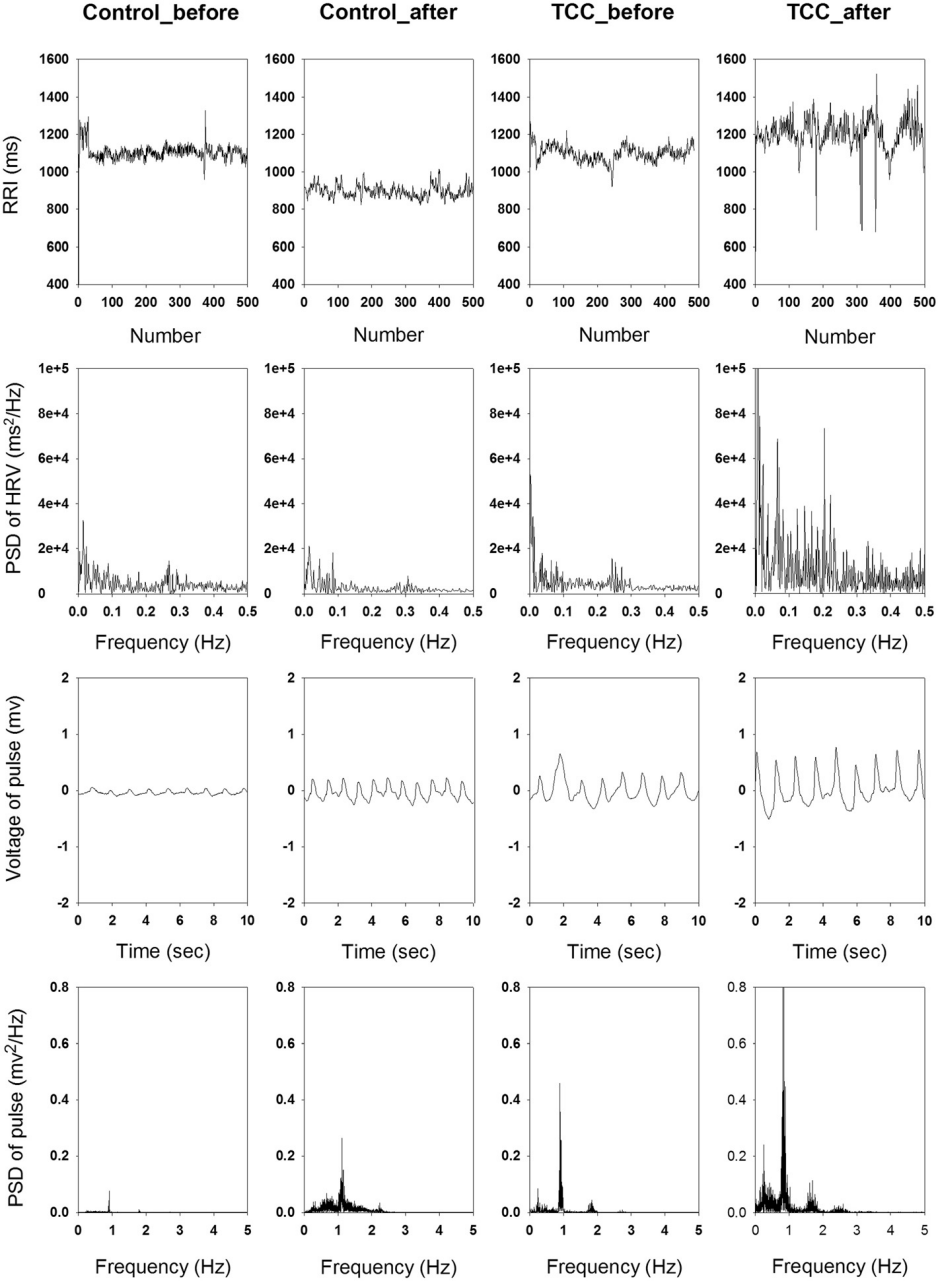
The RRI tachogram, power spectral density of RRI, pulse waveform, and the power spectra of pulse wave in a representative control subject at rest and 30 min after rest, and a representative TCC practitioner at rest and 30 min after TCC exercise

Figure 1 shows the RRI tachogram, power spectrum of RRI, right pulse waveform, and the power spectra of right pulse wave in a representative control subject at rest and 30 min after rest, and a representative TCC practitioner at rest and 30 min after TCC exercise. After TCC, the variation in RRI and the voltage of the pulse wave are increased, as compared with the baseline values. Similarly, the powers of the spectral peaks over all frequency ranges in the HRV spectrum and pulse spectrum are enhanced, as compared with the baseline values.

### Comparison of HRV and pulse harmonics measures between groups at baseline and 30 min after resting or TCC exercise

Table 2 shows that the baseline SD_RR_, CV_RR_, TP, HFP, nLFP and nHFP in the TCC group were all significantly smaller than those of the controls, while the nVLFP and LHR in the TCC group were significantly greater than those of the controls. The baseline TPp, powers of all individual harmonics (Ph1 ~ Ph8), and nPh1 in the TCC group were all significantly greater than those of the controls, while the nPh4 in the TCC group was significantly smaller than that of the controls. This result indicates that the autonomic nervous modulation and pulse wave of the TCC practitioners were different from ordinary healthy people in that the vagal modulation of the TCC practitioners were significantly smaller while the sympathetic modulation of the TCC practitioners were significantly greater than those of the controls. Table 2 also shows that 30 min after rest or TCC exercise, the SD_RR,_ CV_RR,_ TP, ULFP, VLFP, LFP, HFP and nHFP in the TCC group were all significantly smaller than those of the controls, whereas the nVLFP and LHR of the TCC group were significantly greater than those of the controls. However, the TPp, powers of all individual harmonics, nPh1 and nPh2 in the TCC group were significantly greater than those of the controls, while the nPh7 and nPh8 in the TCC group were significantly smaller than those of the controls.

**Table 2:** Comparison of HRV measures and pulse harmonics between control group and TCC practitioners at baseline and 30 min after resting or TCC exercise

Measures	Baseline (n = 30)		30 min after (n = 30)	
*HRV*	*Control*	*TCC*	*Control*	*TCC*
mRRI (ms)	873.8 (756.4–977.2)	917.4 (847.0–992.3)	929.4 (840.2–1082.0)	929.7 (854.8–996.4)
HR (bpm)	68.7 (61.4–79.3)	65.4 (60.5–70.8)	64.6 (55.4–71.4)	64.5 (60.2–70.2)
SD_RR_ (ms)	53.7 (44.3–65.6)	43.8 (29.9–54.5)*	63.7 (53.2–71.5)	40.1 (30.3–52.7)**
CV_RR_ (%)	5.9 (5.2–7.7)	4.4 (3.7–6.0)*	6.8 (5.8–7.1)	4.4 (3.3–5.8)**
TP (ms^2^)	1137 (692–1838)	749 (224–1344)*	17070 (968–2001)	609 (349–1083)**
ULFP (ms^2^)	108 (54–236)	114 (52–167)	195 (116–390)	108 (74–218)**
VLFP (ms^2^)	225 (110–401)	184 (123–305)	273 (196–438)	185.4 (119–213)**
LFP (ms^2^)	360 (187–512)	249 (63–462)	483.8 (292.4–657.9)	200.2 (98.0–367.7)**
HFP (ms^2^)	494 (325–665)	174 (57–582)*	662.0 (453.7–892.6)	178.8 (61.7–484.8)**
nVLFP (nu)	21.0 (15.6–25.9)	31.6 (18.6–55.9)*	21.3 (16.3–26.0)	29.2 (19.9–47.3)**
nLFP (nu)	29.1 (26.1–32.7)	24.5 (12.7–32.3)*	30.0 (27.3–35.6)	33.4 (25.6–39.7)
nHFP (nu)	49.8 (39.9–56.3)	35.2 (25.8–50.6)*	48.5 (39.6–56.1)	31.5 (22.1–45.3)**
LHR	0.61 (0.49–0.89)	1.04 (0.70–2.08)*	0.66 (0.49–0.78)	0.95 (0.62–2.15)**
*Pulse harmonics*	*Control*	*TCC*	*Control*	*TCC*
TPp	3460 (1791–8375)	17775 (12200–22360)*	3089 (818–7648)	19350 (13710–27240)**
Ph1	1741 (889–4346)	12580 (7856–16930)*	1114 (339–4249)	14605 (5554–19090)**
Ph2	301 (187–748)	1504 (1111–2243)*	213 (64–359)	1888 (1006–2680)**
Ph3	65 (33–256)	264 (209–394)*	45 (14–98)	288 (183–475)**
Ph4	20 (7–51)	63 (35–77)*	13 (4–29)	57 (31–90)**
Ph5	7.6 (3.7–18.0)	34.9 (20.6–69.3)*	5.4 (2.4–14.9)	47.2 (27.3–676.0)**
Ph6	1.6 (0.8–9.1)	15.5 (7.1–23.0)*	2.0 (0.9–15.1)	17.2 (8.3–31.2)**
Ph7	1.1 (0.4–3.3)	3.6 (2.4–5.9)*	1.2 (0.4–3.8)	3.3 (2.7–7.0)**
Ph8	0.5 (0.2–1.7)	1.7 (1.1–2.8)*	0.4 (0.2–2.0)	2.1 (0.9–3.2)**
nPh1	41 (30–69)	76 (58–82)*	38 (29–56)	78 (64–83)**
nPh2	9.6 (8.0–14.1)	10.3 (7.9–12.0)	6.5 (3.8–10.8)	10.5 (7.9–11.3)**
nPh3	1.9 (1.6–3.0)	1.7 (1.1–2.3)	1.8 (0.9–2.9)	1.6 (1.2–2.8)
nPh4	0.6 (0.3–1.0)	0.4 (0.2–0.5)*	0.4 (0.2–0.8)	0.3 (0.2–0.5)
nPh5	0.2 (0.2–0.4)	0.3 (0.2–0.4)	0.2 (0.1–0.4)	0.2 (0.2–0.3)
nPh6	0.06 (0.03–0.19)	0.11 (0.05–0.14)	0.1 (0.1–0.2)	0.1 (0.5–0.2)
nPh7	0.03 (0.01–0.08)	0.02 (0.01–0.04)	0.16 (0.02–0.09)	0.02 (0.01–0.04)**
nPh8	0.01 (0.01–0.04)	0.01 (0.01–0.02)	0.02 (0.01–0.05)	0.01 (0.01–0.02)**
F1 (Hz)	1.1 (1.0–1.3)	1.1 (1.0–1.2)	1.1 (0.9–1.2)	1.1 (1.0–1.2)

*p < 0.05 vs. control group at baseline; ** *p* < 0.05 vs. control group 30 min after resting

Values are numbers of patients or medians (25 % ~ 75 %). *mRRI* mean RR intervals; *HR* heart rate; *SD*_*RR*_ standard deviation of RR intervals; *CV*_*RR*_ coefficient of variation of RR intervals; *TP* total power; *ULFP* ultra low-frequency power; *VLFP* very low-frequency power; *LFP* low-frequency power; *HFP* high-frequency power; *nVLFP* normalized very low-frequency power; *nLFP* normalized low-frequency power; *nHFP* normalized high-frequency power; *LHR* low-/high- frequency power ratio; *TPp* total power of pulse in the power spectrum; *nPhx* normalized power of harmonic x; *F1* frequency of the 1^st^ harmonic

Correlation coefficients between TCC age and HRV measures and pulse harmonics in the TCC practitioners were also calculated, and we found that the TCC age (7.0 ± 8.2 years) correlated significantly and negatively with the baseline nPh1 and nearly significantly and negatively with the baseline nPh2 in the TCC group.

### Comparison of percentage changes in HRV and pulse harmonics measures between groups

Table 3 compares the percentage changes in HRV measures and pulse harmonics 30 min after resting or TCC exercise between 2 groups of subjects. In the HRV, the %mRRI, %SD_RR_, %TP, and %VLFP were significantly decreased while the %HR, %ULFP and %nLFP were significantly increased in the TCC practitioners, as compared with the controls. In the pulse harmonics, only the %Ph2 and %F1 were significantly increased after TCC in the TCC practitioners, as compared with the controls. These results suggest that TCC exercise can decrease the vagal modulation and increase the sympathetic modulation of the subjects. The meaning and significance of the increase in %nPh2 and %nLFP after TCC deserve further considerations.

**Table 3:** Comparison of the percentage change in HRV measures and pulse harmonics 30 min after resting or TCC exercise between the control and TCC groups

**Measures**	Control (n = 30)	TCC (n = 30)	P value
*HRV*			
%mRRI (%)	8.7 (5.8 − 14.9)	2.2 (-0.5 − 6.3)	<0.001
%HR (%)	−8.0 (-13.0 − -5.6)	−2.2 (-5.9 − 0.5)	<0.001
%SD_RR_ (%)	14.1 (-1.1 − 27.5)	1.0 (-6.7 − 6.8)	0.014
%CV_RR_ (%)	2.9 (-4.5 − 15.6)	−0.5 (-8.7 − 6.5)	0.206
%TP (%)	25.7 (-4.3 − 80.8)	2.9 (-22.1 − 29.1)	0.010
%ULFP (%)	−72.4 (-88.6 − -42.2)	8.7 (-34.7 − 164.1)	<0.001
%VLFP (%)	30.7 (-5.7 − 97.4)	−17.4 (-35.8 − 32.3)	0.016
%LFP (%)	29.1 (3.4 − 66.4)	4.5 (-28.5 − 65.3)	0.077
%HFP (%)	29.7 (5.7 − 65.6)	16.2 (-18.0 − 70.6)	0.137
%nVLFP (%)	4.9 (-11.5 − 18.6)	1.8 (-24.4 − 26.5)	0.579
%nLFP (%)	−3.0 (-11.3 − 18.7)	34.3 (-3.5 − 195.7)	0.018
%nHFP (%)	0.6 (-10.7 − 11.6)	−8.8 (-38.4 − 25.2)	0.258
%LHR (%)	−4.8 (-18.0 − 36.4)	−4.5 (-35.0 − 26.8)	0.340
*Pulse harmonics*			
%TPp	−32.9 (-72.7 − 272.6)	17.9 (-21.0 − 39.2)	0.579
%Ph1	−39.4 (-78.4 − 262.7)	17.3 (-18.1 − 54.0)	0.186
%Ph2	−59.6 (-79.5 − 21.5)	15.3 (-22.4 − 29.4)	0.030
%Ph3	−27.1 (-70.0 − 71.3)	16.2 (-14.9 − 72.2)	0.176
%Ph4	−31.4 (-76.0 − 131.4)	26.1 (-40.8 − 65.6)	0.217
%Ph5	−11.3 (-63.3 − 165.7)	0.3 (-30.7 − 56.9)	0.620
%Ph6	7.3 (-52.6 − 526.3)	22.1 (-27.6 − 108.8)	0.842
%Ph7	−18.3 (-53.0 − 172.9)	1.7 (-33.4 − 85.0)	0.877
%Ph8	20.6 (-59.2 − 376.4)	2.0 (-43.5 − 78.2)	0.355
%nPh1	−17.8 (-51.3 − 32.7)	1.8 (-5.8 − 5.0)	0.085
%nPh2	−26.8 (-67.5 − 10.8)	−2.1 (-15.5 − 17.4)	0.072
%nPh3	−20.0 (-67.3 − 34.8)	5.4 (-24.7 − 38.0)	0.212
%nPh4	−8.3 (-59.6 − 68.7)	2.9 (-30.0 − 23.1)	0.420
%nPh5	−4.0 (-56.7 − 105.0)	7.4 (-30.0 − 23.9)	0.807
%nPh6	39.5 (-41.5 − 217.5)	6.7 (-35.1 − 54.3)	0.196
%nPh7	63.8 (-28.3 − 111.6)	0.5 (-41.4 − 58.5)	0.206
%nPh8	−0.8 (-41.2 − 229.7)	−4.7 (-38.4 − 32.6)	0.340
%F1	−7.8 (-13.8 − -4.4)	−2.7 (-7.4 − 2.9)	0.002

Values are numbers of patients or medians (25 % ~ 75 %)

### Correlation between percentage changes in HRV measures and percentage changes in pulse harmonics

Table 4 shows that the %SD_RR_ and %CV_RR_ correlate significantly and negatively with the %nPh1, and that the %CV_RR_ correlate significantly and negatively with %nPh8 in the control group. This result suggests that the power of the 1^st^ and 8^th^ harmonics might have inverse relationship with the vagal modulation of the subjects. In the TCC group, the %HR correlates significantly and positively with the %TPp, %Ph1, %Ph2, and %F1, and significantly and negatively with %Ph7, and %nPh6 to %nPh8. Furthermore, the %SD_RR_ correlates significantly and positively with %Ph3, %Ph6, and %Ph7, and significantly and negatively with %F1. These results suggested that an increase in HR or a decrease in vagal modulation due to TCC exercise might be associated with an increase in the TPp and the powers of the 1^st^ and 2^nd^ pulse harmonics, and a decrease in the powers of 6^th^ to 8^th^ pulse harmonics in the TCC group.

**Table 4:** Correlation coefficients between the percentage change in time and frequency domain HRV measures and the percentage change in pulse harmonics in both groups of subjects

Measures	%mRRI	%HR	%SD_RR_	%CV_RR_	%TP	%ULFP	%VLFP	%LFP	%HFP	%nVLFP	%nLFP	%nHFP	%LHR
*Control group*													
%TPp	−0.05	0.04	−0.12	−0.12	−0.11	−0.13	−0.14	−0.11	−0.05	−0.14	−0.08	0.10	−0.13
%Ph1	−0.01	−0.01	0.15	−0.16	−0.13	−0.09	−0.16	−0.14	−0.07	−0.14	−0.11	0.12	−0.16
%Ph2	−0.06	0.05	−0.13	−0.12	−0.13	−0.10	−0.14	−0.14	−0.07	−0.11	−0.10	0.11	−0.15
%Ph3	0.01	−0.03	−0.02	−0.02	−0.08	0.13	−0.08	−0.11	−0.02	−0.04	−0.14	0.06	−0.15
%Ph4	−0.05	0.02	0.18	0.22	−0.01	0.33	−0.03	−0.11	0.01	0.01	−0.05	−0.02	−0.05
%Ph5	0.18	−0.18	0.18	0.15	−0.05	0.44*	0.07	0.04	0.04	0.12	−0.05	−0.11	−0.01
%Ph6	0.28	−0.28	0.31	0.23	0.12	0.57*	−0.01	0.12	0.16	−0.12	0.02	−0.05	0.02
%Ph7	0.19	−0.19	0.09	0.03	−0.07	0.40*	−0.14	−0.01	0.01	−0.15	−0.08	0.05	−0.13
%Ph8	0.22	−0.24	0.28	0.23	0.14	0.44*	−0.01	0.18	0.14	−0.13	0.03	−0.07	0.05
%nPh1	0.13	−0.12	−0.37*	−0.46*	−0.31	−0.08	−0.17	−0.18	−0.35	0.05	0.19	0.15	−0.01
%nPh2	−0.13	0.12	−0.34	−0.32	−0.30	−0.19	−0.24	−0.14	−0.29	−0.19	0.43*	0.16	0.08
%nPh3	−0.03	0.03	−0.16	−0.16	−0.17	−0.01	−0.09	−0.19	−0.20	0.07	−0.15	−0.03	−0.13
%nPh4	−0.12	0.11	0.13	0.21	−0.08	0.35	−0.02	−0.03	−0.15	0.12	0.13	−0.14	0.11
%nPh5	−0.02	0.01	−0.15	−0.15	−0.16	−0.02	−0.09	−0.10	−0.18	0.01	0.16	0.01	0.02
%nPh6	0.33	−0.32	0.11	−0.01	0.10	0.14	0.03	0.08	0.21	−0.14	0.22	0.06	0.03
%nPh7	0.03	−0.04	−0.27	−0.31	−0.21	−0.17	−0.26	−0.17	−0.13	−0.35	0.06	0.19	−0.13
%nPh8	0.05	−0.07	−0.32	−0.38*	−0.24	−0.18	−0.23	−0.22	−0.23	−0.16	−0.12	0.13	−0.18
%F1	−0.71*	0.72*	−0.12	0.16	−0.29	0.03	−0.17	−0.22	−0.41*	0.18	−0.01	−0.09	0.04
*TCC group*													
%TPp	−0.42*	0.48*	−0.06	0.09	−0.06	−0.10	−0.001	−0.06	−0.10	0.08	−0.11	0.01	0.04
%Ph1	−0.43*	0.49*	−0.06	0.09	−0.06	−0.10	−0.003	−0.06	−0.11	0.08	−0.10	−0.01	0.05
%Ph2	−0.42*	0.48*	−0.03	0.13	−0.03	−0.03	0.02	−0.06	−0.06	0.07	−0.05	−0.02	−0.01
%Ph3	0.23	−0.19	0.40*	0.35	0.38*	0.24	0.41*	0.08	0.32	0.15	−0.06	−0.03	−0.20
%Ph4	0.08	−0.07	0.31	0.30	0.31	0.10	0.28	0.22	0.19	0.02	0.00	−0.05	0.07
%Ph5	−0.14	0.19	0.20	0.26	0.22	0.04	0.26	0.05	0.11	0.12	−0.18	0.02	−0.05
%Ph6	−0.26	−0.21	0.47*	0.41*	0.48*	0.21	0.48*	0.15	0.32	0.13	−0.19	−0.008	−0.10
%Ph7	−0.45*	−0.40*	0.44*	0.31	0.41*	0.27	0.40*	0.14	0.30	0.04	−0.16	0.006	−0.10
%Ph8	−0.17	−0.12	0.24	0.19	0.21	0.16	0.14	0.08	0.27	−0.08	−0.05	0.004	−0.13
%nPh1	−0.22	0.20	−0.11	−0.03	−0.10	−0.11	−0.07	−0.03	−0.24	0.03	0.003	−0.18	0.34
%nPh2	−0.19	0.17	−0.16	−0.09	−0.15	0.05	−0.12	−0.10	−0.17	0.05	0.15	−0.23	0.13
%nPh3	−0.25	−0.25	0.07	−0.01	0.05	0.31	0.15	−0.20	0.02	0.24	−0.02	−0.19	−0.25
%nPh4	−0.15	0.13	0.02	0.10	0.01	−0.01	−0.06	0.14	−0.12	−0.13	0.02	−0.13	0.45*
%nPh5	−0.18	0.17	−0.03	0.05	−0.03	−0.01	−0.06	−0.03	−0.17	−0.06	−0.12	−0.11	0.28
%nPh6	−0.48*	−0.48*	0.23	0.06	0.22	0.33	0.26	−0.12	0.16	0.13	−0.20	−0.07	−0.34
%nPh7	−0.45*	−0.43*	0.24	0.09	0.20	0.32	−0.21	−0.03	0.15	0.06	−0.14	−0.02	−0.18
%nPh8	−0.37*	−0.37*	0.13	0.01	0.06	0.33	−0.05	−0.06	0.15	−0.17	−0.08	−0.03	−0.16
%F1	−0.76*	0.76*	−0.46*	−0.22	−0.40*	−0.25	−0.46*	0.12	−0.38*	−0.17	0.11	0.09	0.43*

* *P* < 0.05

Table 4 also shows that the %HFP also correlated significantly and negatively with %F1 in both groups. The %TP of HRV correlates significantly and positively with %Ph3, %Ph6, %Ph7, and %F1 in the TCC group only. The LHR also correlates significantly and positively with %nPh4 and %F1 in the TCC group only. These results suggest that an increase in sympathetic modulation due to TCC exercise might be associated with the increased HR and nPh4 in the TCC group.

## Discussion

This study investigated the autonomic nervous modulation and pulse harmonics of TCC practitioners, and investigated the associations between the changes in pulse harmonic and the autonomic nervous modulation after TCC exercise in TCC practitioners. Our results showed that the baseline autonomic nervous modulation and pulse harmonics of TCC practitioners are already different from those of ordinary healthy people because the vagal modulation of the TCC practitioners is suppressed while the sympathetic modulation of the TCC practitioners is enhanced, and the powers of all pulse harmonics are greatly increased except a slight dip in the power of the 4^th^ pulse harmonics, as compared with the healthy controls. After TCC exercise, the vagal modulation was decreased and the sympathetic modulation and the power of the 2^nd^ harmonics were increased.

It was unexpected that the baseline vagal modulation of the TCC practitioners were found to be significantly smaller while the sympathetic modulation of the TCC practitioners were significantly greater than those of the controls, because the vagal modulation is known to be decreased in many kinds of pathological conditions, such as aging [32], acute myocardial infarction [33], diabetes mellitus [34], chronic renal failure [35], congestive heart failure [36], and chronic obstructive pulmonary disease [37]. It has been demonstrated that Tai chi (TC) is feasible and safe in heart failure with preserved ejection fraction (HFPEF), and therapeutic endpoints appear similar with TC relative to aerobic exercise despite a lower aerobic training workload [38]. However, in view of the fact that the baseline TPp, powers of all individual harmonics (Ph1 ~ Ph8) and nPh1 in the TCC group were all significantly greater than those of the controls, the findings of suppressed vagal modulation and enhanced sympathetic modulation in TCC practitioners might be comprehensible because the changes in pulse harmonics in TCC practitioners might be caused by the changes in their autonomic nervous modulation.

The pulse plethysmography was found to be useful in the monitoring of vascular sympathetic tone [39, 40], and can be changed dramatically in cardiovascular shock [41] and sedation [42]. Thus, the pulse waveform analysis might be used as a non-invasive measure of peripheral vascular responsiveness, with potential utility in the evaluation of various kinds of cardiovascular diseases [43]. McDonald [28] first performed the Fourier transform of the arterial pulse waves, and found that the frequency of each harmonic is the multiple of the heart rate which is the fundamental frequency, and explained the phenomenon of the difference between pressure waves in central and peripheral arteries on the basis of wave reflection. It was later found that the ratio of energy distribution in different harmonic bands can be determined by the spectral harmonic energy ratio, and this method can give us a novel viewpoint from which to comprehend and quantify the spectral harmonic distribution of circulation information conveyed by the arterial pulse [44]. According to the resonance theory of Wang et al. [14, 16, 17], the aorta and the attached organ can produce coupled oscillation that is analogous to a resonance circuit, and that the physical conditions of the internal organs or tissues will influence the resonant status and the distributions of blood pressure. By analyzing the variation in pulse spectrum after organ ligation, acupuncture and herbs in the rats, the resonance frequencies of the liver, kidney, spleen, lung, and stomach of the rats were found to be related to the 1^st^, 2^nd^, 3^rd^, 4^th^ and 5^th^ harmonics in pulse spectrum, respectively [45–47]. In this study we found that the power of the nPh1 of the TCC group before TCC was significantly higher than that of controls, whereas the power of the nPh4 of the TCC group was significantly lower than that of controls (Table 2). The TCC age correlated significantly and positively with the baseline nPh1, and nearly significantly with the baseline nPh2 of the TCC practitioners (Table 3). We also found that 30 min after TCC the nPh1 and nPh2 of the TCC group were significantly higher than those of controls, whereas the power of the nPh7 and nPh8 were significantly lower than those of controls (Table 4). By referring to the resonance theory of Wang et al [15, 16], the changes in the power spectrum of the pulse observed in this study might be caused by the changes in the blood flow to the vital organs attached to the aorta. Whether or not this is true requires future studies to verify.

Comparison of the percentage changes in pulse harmonics showed that only the %Ph2 was increased in the TCC group after TCC exercise relative to the controls in this study (Table 3). Correlation analysis shows that the %Ph2 correlates significantly and negatively with %mRRI and significantly and positively with %HR in the TCC practitioners (Table 4). These findings are similar to the finding of Su et al. [18] that the power of the 2^nd^ pulse harmonic was increased, whereas the total power of pulse and the powers of higher order harmonics were decreased during pregnancy. Decreased vascular resistance due to pregnancy was proposed to account for the increased power of the 2^nd^ pulse harmonic during pregnancy [18]. Thus, the observed increase in the power of the 2^nd^ pulse harmonic in TCC practitioners might also be caused by the decreased vascular resistance after TCC exercise. If this speculation is true, then it can be understood why TCC exercise is good for the health of the cardiovascular system. Further researches are needed to clarify the meaning of the changes in pulse harmonics after TCC exercise.

## Conclusion

In conclusion, TCC practice does have an effect on the autonomic nervous modulation and pulse harmonics of its practitioners. The TCC practitioners have increased total power of pulse and the powers of the 1^st^ and 2^nd^ pulse harmonics, and decreased power of the 4^th^ pulse harmonics, along with decreased vagal modulation and increased sympathetic modulation. After TCC exercise, the power of the 2^nd^ harmonics is increased which might be related to the increased HR due to decreased vascular resistance after TCC exercise.
